# Radial Basis Function Neural Network-Based Modeling of the Dynamic Thermo-Mechanical Response and Damping Behavior of Thermoplastic Elastomer Systems

**DOI:** 10.3390/polym11061074

**Published:** 2019-06-21

**Authors:** Ivan Kopal, Marta Harničárová, Jan Valíček, Jan Krmela, Ondrej Lukáč

**Affiliations:** 1 Alexander Dubček University of Trenčín, Faculty of Industrial Technologies in Púchov, Ivana Krasku 491/30, 020 01 Púchov, Slovakia; ivan.kopal@fpt.tnuni.sk (I.K.); jan.krmela@fpt.tnuni.sk (J.K.); 2 Slovak University of Agriculture in Nitra, Technical Faculty, Tr. A. Hlinku 2, 949 76 Nitra, Slovakia; jan.valicek@uniag.sk (J.V.); ondrej.lukac@uniag.sk (O.L.); 3 Institute of Technology and Business in České Budějovice, Faculty of Technology, Department of Mechanical Engineering, Okružní 10, 370 01 České Budějovice, Czech Republic

**Keywords:** artificial neural networks, radial basis functions, thermoplastic polyurethanes, visco-elastic properties, dynamic mechanical analysis

## Abstract

The presented work deals with the creation of a new radial basis function artificial neural network-based model of dynamic thermo-mechanical response and damping behavior of thermoplastic elastomers in the whole temperature interval of their entire lifetime and a wide frequency range of dynamic mechanical loading. The created model is based on experimental results of dynamic mechanical analysis of the widely used thermoplastic polyurethane, which is one of the typical representatives of thermoplastic elastomers. Verification and testing of the well-trained radial basis function neural network for temperature and frequency dependence of dynamic storage modulus, loss modulus, as well as loss tangent prediction showed excellent correspondence between experimental and modeled data, including all relaxation events observed in the polymeric material under study throughout the monitored temperature and frequency interval. The radial basis function artificial neural network has been confirmed to be an exceptionally high-performance artificial intelligence tool of soft computing for the effective predicting of short-term viscoelastic behavior of thermoplastic elastomer systems based on experimental results of dynamic mechanical analysis.

## 1. Introduction

### 1.1. Thermoplastic Elastomers

Thermoplastic elastomers (TPEs) are high molecular weight polymeric materials that combine the mechanical properties of vulcanized elastomers (rubbers) with excellent processability and recyclability of thermoplastics [1]. Thermoplastics are composed of long linear macromolecular chains that are bound to each other by weak van der Waals forces. These intermolecular interactions significantly weaken with increasing temperature. As a result of the more intense thermal movement of macromolecules, thermoplastic softens and can repeatedly liquefy when the temperature continues to rise. Therefore, thermoplastics belong to a well thermoformable and easy to recycle polymers [2]. On the other hand, vulcanized elastomers are characterized by strong covalent bonds forming nodes of a spatial polymer network that are so strong that they cannot be disrupted without irreversibly degrading the original material through an increase in temperature. Also, it is this property that makes them difficult-to-recycle and non-eco-friendly plastics without any possibility of multiple heat treatment [3]. 

TPEs are polymers with a biphasic structure formed by mutually immiscible hard and soft segments (domains) [1]. Soft segments provide high elasticity, while hard segments act as chemical bonds and fillers in rubbers that limit the mobility of soft segments. The soft phase is usually represented by polybutadiene, poly(ethylene-co-alkene), polyisobutylene, poly(oxyethylene), poly(ester), polysiloxane or any of the typical elastomers. The solid phase can be composed of polystyrene, poly(methyl methacrylate), urethane, ionomer – poly(ethylene-co-acrylic acid) (sodium, Mg, Zn salt), ethylene propylene diene monomer, or fluoropolymers [4]. The thermoplastic hard domains dispersed in the continuous elastomeric phase form nodes of the spatial TPE network. In conventional polymer blends at the interface of two phases, relatively small forces act, but in the case of TPEs, strong physical bonds are formed at the phase-to-phase interface with energy that are comparable to that of covalent bonds. The thermoplastic domains in TPEs are considerably longer than the chemical bonds forming nodes of the traditional polymer network in the rubbers; therefore, they also act as a filler and can have a favorable reinforcing effect on their mechanical properties. The thermoplastic domains of TPEs are solid at room temperature and join the elastomeric chains into a three-dimensional physical network. However, when heated above the melting point, the rigid segments soften, the physical network disintegrates, TPE passes into a flowable melt and undergoes processing using conventional plastic technologies [5]. After cooling, the thermoplastic domains are re-grouped, hardened and form rigid nodes of the physical network without altering the chemical structure of the parent polymer, thereby returning TPE to the original properties of the source plastic. Compared to rubbers, TPEs have comparable utility properties without the need for energy, time and cost-intensive processes of chemical or ecologically more acceptable and cheaper elastomers vulcanization by ionizing radiation [6], which is one of their major technological advantages. The TPE properties depend on the number of internal and external factors, in particular temperature, pressure and the frequency of the external dynamic mechanical load, which is the most common way of stressing any materials in practical applications [7].

### 1.2. Thermoplastic Polyurethanes

Thermoplastic polyurethanes (TPUs) are also included in the broad TPEs family in terms of the chemical structure of polymer macromolecules. They represent a unique category of plastics created when polyaddition reaction occurs between a diisocyanate and one or more diols [8]. Like all multi-block copolymers of TPEs, TPUs also represent a high molecular weight linear segmented semi-crystalline multi-block copolymers with a biphasic structure formed by alternating microphase-separated, mutually immiscible short rigid crystalline domains (hard segments) and longer soft amorphous flexible chains (soft segments). Highly polarized hard segments tend to be composed of low molecular weight glycols, or diamine reacted with a diisocyanate, and provide the resulting material with strength. Low polarized soft segments with a relatively high molecular weight can be composed of polyester or polyether macromolecules [8]. The high polarity of the hard segments causes a strong, attractive interaction between them, resulting in a high degree of aggregation and arrangement in the solid phase, or the forming of crystalline and pseudo-crystalline domains dispersed in a soft, flexible matrix. As a result of this phase separation, due to mainly the thermodynamic immiscibility or incompatibility between the hard and soft phase, hard segments are dispersed as micro-domains, only tens of nanometres in size, in the TPU’s structure and both types of hard and soft segments. These segments are linked together by strong covalent links, so that they actually form block copolymers. Nevertheless, the level of this phase separation depends mainly on the degree of polarity and molecular weight of the flexible chains, the production conditions and many other factors. In addition, crystallization may also occur in the soft phase of TPU [9]. The crystalline and pseudo-crystalline areas operate as physical cross-links that are responsible for high elasticity of TPUs, whereas the flexible soft domains determine their elongation characteristics. However, these physical cross-links disintegrate rapidly under the effect of heat so TPUs can be well processed by traditional processing methods. They are quite easily thermally reprocessed and recyclable, which makes them highly cost-effective for a variety of practical applications [10]. The unique chemical structure of TPUs described above results in the possibilities of a large number of combination of very interesting properties [11]. Therefore, TPUs have long been a subject of very intensive research from an industrial, as well as an academic point of view.

### 1.3. Viscoelastic Behavior of Thermoplastic Polyurethanes

The properties of all polymers vary substantially with various perturbations. The vast majority of them are especially dependent on temperature, which is responsible for different physical states of polymers and induces particular relaxation transition processes at different values [12], due to different conformational changes in the polymer chains, as well as the morphological and structural changes in the polymer, that determine their behavior at various conditions. In addition, virtually all physical properties of polymers depend not only on temperature but also on time. The time dependency in the case of mechanical properties is determined by the nature of polymer response to external mechanical loading [12].

Generally, the mechanical response of all real polymers including TPUs is viscoelastic, meaning that their solid state deformation is recoverable, time-dependent and disappears immediately or sometime after unloading. While the temperature primarily affects the viscous component of the viscoelastic response (viscous flow or thermodynamically irreversible deformation), its elastic component (elasticity or thermodynamically reversible deformation), through the effect of a deformation rate, long-term relaxations (stress relaxation and creep) as well as short-term relaxations (dynamic storage and loss moduli), is a more or less complicated function of time [13]. The presented work is devoted to modeling of short-term relaxation processes and prediction of short-term viscoelastic properties of TPUs based on experimental results of dynamic mechanical analysis (DMA), which is one of the most sensitive test and analytical methods used in the study of viscoelastic behavior of polymeric materials and thermal relaxation events occurring under different conditions of dynamic mechanical loading. 

### 1.4. Dynamic meChanical Analysis of Polymers

In the DMA test, a weak sinusoidally varying stress is applied at various frequencies, and the strain inside a tested sample of polymeric material with precisely defined geometry is recorded as temperature increases. The test speed or time scale used for cyclically deforming the material enables the study of time (or frequency) effects on its resistance to permanent deformation [14]. The generally known fact is taken into account that a dynamic mechanical experiment carried out at a frequency *ω* is qualitatively equivalent to the experiment performed in the time domain *ω*^−1^. Information obtained through the strategic use of the three parameters of applied force with a very small amplitude, temperature, and frequency (or time) provides the basis for predicting polymer performance in real-world applications. DMA measurements usually result in temperature and/or frequency dependencies of the storage (elastic) modulus *E′*, loss (viscous) modulus *E″* (as a real and imaginary component of complex dynamic modulus E^*^, respectively) and loss tangent tan*δ*. They make it possible to quantify the amount of elastic energy stored in the material, the amount of energy dissipated into heat under dynamic mechanical load and the mechanical damping or the ratio of loss and storage modulus, respectively. Due to the viscoelastic nature of the polymers, the sinusoidal strain lags the applied stress by a phase angle *δ*, which is a measure of its viscoelastic damping or internal molecular friction [14].

Temperature dependencies of dynamic thermo-mechanical parameters *E′*, *E″* and tan*δ* allow us to identify the thermal transitions in and between different physical states of polymers – the secondary transitions in glassy stage and the primary transitions from glassy-to-leathery stage (glass transition), from leathery-to-rubbery stage and from rubbery-to-decomposed stage, which take place at the corresponding transition temperatures. The glass transition temperature *T*_g_ or *T*_α_, crystalline melt temperature (or melting point) *T*_m_ and flow temperature *T*_f_ [12] are characteristic of semi-crystalline thermoplastics, non-crosslinked elastomers and thermoplastic elastomers, while the crosslinked polymers up to the temperature of degradation *T*_d_ only show the glass transition temperature. At very low temperatures in glassy stage, secondary gamma transition can also be observed at temperature *T*_γ_ and beta transition at temperature *T*_β_ due to localized bond movements (bending and stretching) and side chain movements, as well as by the whole side chains and localized groups of 4–8 backbone atoms movements, respectively. The beta transition at temperature *T*_β_ often represents the *T*_g_ of a secondary component in a blend or a specific block in the block copolymers such as TPUs [15]. The secondary delta transition at temperature *T*_δ_ is related to very small motions within macromolecule at temperatures under *T*_γ_. Above the glass transition temperature, crystalline and semi-crystalline polymers may pass through T^*^ and *T*_II_ transition temperatures, indicating a slippage of the crystallites past each other and a movement of coordinated segments in the amorphous phase at higher temperatures that relate to reduced viscosity [16]. 

All relaxation transitions mentioned above are frequency dependent. With increasing the frequency of dynamic mechanical loading, the transition temperatures of relaxation events, in accordance with the time-temperature-frequency superposition principle [14], shift to higher values. The temperature shift allows the frequency dependence of dynamic thermo-mechanical parameters to determine the activation energies, which are necessary to overcome the potential energy barriers preventing the respective type of relaxation movement of the polymer macromolecules [17].

### 1.5. Artificial Neural Networks Modeling

Many different mathematical models describing the viscoelastic response of polymers to external mechanical loading have been developed over the years. However, only the unified physically-based statistical stiffness–temperature, stiffness–frequency, and stiffness–strain rate models, presented by Mahieux and Reifsnider and Richeton et al. in the works [18,19], seem to be valid over a wide range of polymeric materials, frequencies, and strain rates in the whole temperature range throughout their service life. Moreover, they can also reliably predict all of the thermal relaxation transitions occurred in the various polymeric systems from the glassy up to the flow stage. More detailed information about the physical nature of these models, based on Weibull distribution of the breakage of secondary bonds during the relaxation processes in polymers, can be found, e.g., in our earlier works [17,20,21]. The problem of successful modeling of the short-term viscoelastic response of polymeric systems lies mainly in the complex and strongly nonlinear nature of temperature dependencies of dynamic thermo-mechanical parameters *E′*, *E″* and tan*δ*, which considerably complicates their analytical expression by traditional mathematical methods. However, where common model-driven approaches fail, one of the data-driven approaches of so-called soft computing should be applied, such as Technique of Artificial Neural Network Modeling. This technique can directly cover all the complicating effects of the addressed problem without needing to understand its physical nature. It allows us to search for relationships between the input-output parameters of the corresponding model by learning from examples of relationships between appropriately selected representative experimental data [22]. Since it is well known that artificial neural networks are an extremely powerful universal approximator for random functional dependencies between any number of variables [23], it is expected that temperature and frequency dependencies *E′*(*T*, *f*), *E″*(*T*, *f*) and tan*δ*(*T*, *f*) could be successfully modeled by them.

The concept of artificial neural networks (ANNs) is an information-processing paradigm inspired by human brain capabilities to store and process the complex information. It was designed to study the behavior of the real, non-linear, static-dynamic, complex systems by computer simulation via the recognition, classification and generalization of learned patterns. A structure of the ANN’s is composed of a large number of highly interconnected and mutually interacting processing elements (artificial neurons) organized in layers analogous to biological neurons that are tied together with weighted connections (synaptic weights) analogous to biological synapses [24]. Each ANN consists of at least three layers – one input layer, at least one hidden layer, and one output layer. Each neuron of the ANN receives inputs either from many other neurons or from an external stimulus, changes its internal state (activation) correspondingly, and produces output depending on the input and activation, which is sent to the input of other network neurons or its output. The topology or structure of an ANN defines how the neurons in different layers are connected, which determines how they exchange information during its application. The synaptic strength of the connection between neurons is evaluated by the weights representing the probability of data transmission along with a path connection. The choice of the topology of the ANN to be used closely depends on the requirements of the concrete solutions to the problem. The adaptive weights along paths between connected neurons and the functions that compute the neuron activation (activation functions) can be tuned by a learning algorithm that learns from observed data in order to design the ANN’s model of any complex relationships between them even if no functional model exists [25]. Generally, the main feature of neural networks is the ability to abstract relationships between the values of independent (input) and dependent (output) variables presented in a suitable form (in the shape of vectors), and the subsequent application of rules obtained by this abstraction (“black box” model) to any input values. The relationship abstraction process implemented on a set of input-output data (patterns) is a network learning (training) process during which the neuron weighting values are modified by the learning algorithm until the optimality criterion determined by the cost function (usually by a Mean squared error), ensuring that network global error minimization is met. After the learning process is completed, the weight values are no longer changed, and the trained network produces (simulates) outputs based on the found rules applied to any input data coming from the same distribution, which corresponds to the generalization process of the acquired knowledge stored in the weights. It is this collection of weights that is called an ANN model because this collection of weights represents an attempt to guess the relationship between the input-output data patterns and correctly grasp their basic structure [26].

The issue of ANNs is discussed in detail in a number of publications, such as the monograph [27], or in our works [28,29,30], where there is a significant space devoted to various learning algorithms of various ANNs types. Therefore, only the type of ANN will be mentioned here, namely the radial basis function ANN, which is directly related to the solution of the presented work issue, i.e. to the approximation of multi-variable functional dependencies of experimental data. 

### 1.6. Radial Basis Function Artificial Neural Network

The radial basis function artificial neural network (RBF-ANN) is a three-layer ANN consisting of an input layer, a single hidden layer, containing RBF neurons, and an output layer, containing linear or non-linear neurons. The transfer function of hidden RBF neurons is an attenuation, central-radial symmetry, non-negative, non-linear function. All functions that depend only on the distance from a center vector are radially symmetric about that vector, hence the name radial basis function [24]. Usually, the hidden layer neurons include the non-linear RBF Gaussian function, and a non-linear sigmoid or linear function is located in output layer neurons as its transfer (activation) function. The linear output layer transfer function is used for function approximation problems [31]. In this type of ANN architecture, each neuron is connected to all of the neurons in the next layer, but there are no connections between the neurons on the same layer (fully connected architecture). The information brought into the input layer, which performs no transfer function on the input signal and only transfers input data to the network from the environment, propagates through the hidden layer in a forward direction to its output layer (feed-forward ANN structure) [27]. The information transferred from linear input layer to non-linear hidden layer is processed in such a way that output of each hidden neuron is inversely proportional to the Euclidean distance from the input vector to the center of the RBF of this neuron. In the case of Gaussian activation function, the weights of hidden layer neurons represent the center of the symmetrical Gaussian bell curve. These weights are predetermined in such a manner that the entire input space is covered by the field of Gaussian activation functions and the weights of synaptic connections between hidden and output neurons are searched for in the network learning process [32]. The output layer computes the sum of weights and biases of all the RBFs outputs. The development and use of RBF-ANN involves determining the optimal number of hidden neurons, the centercenters and width of the RBFs (which is a measure of the spread of patterns), as well as the weights for the output layer. A bias value allows for shifting the activation function to the left or right, which may be critical for successful learning, and it helps to get a better fit for the data or a better prediction function as ANN output [33]. At each iteration of a learning algorithm, the position of the RBF centercenters, its width and the linear weights to each output neuron are modified. The learning process is completed when each RBF center is brought up as close as possible to the input vector, and the error of the network’s output is within the desired limit. So, the approximation of any functional dependence between variables may be expressed as a linear combination of an optimal number of suitably positioned RBFs with adequate width [32]. The RBF-ANN is also an error-back-propagation ANN. However, during the learning, all of the inputs of RBF-ANN are fed to the hidden layer directly without any weight, and only the weights between hidden and output layer have to be modified using an error signal. Therefore, RBF-ANN requires a much shorter learning time in comparison to multi-layer feed-forward back-propagation ANNs, which are the most widely used ANNs in all types of practical applications [27], although their creation is considerably more demanding than the creation of RBF-ANNs.

Generally, there is currently only a relatively modest number of works, e.g. [34,35,36], which deal with the application of RBF-ANN in the field of modeling the physical properties of polymers and predicting their behavior under different conditions. In this work, RBF-ANN with Gaussian activation function was applied in the modeling and prediction of dynamic thermo-mechanical response and damping behavior of TPEs based on complex results of DMA tests of TPU.

## 2. Materials and Methods 

### 2.1. Sample Preparation

The synthesis of the TPU under investigation was performed via a one-shot procedure [37] by mixing the commercial PX 522/HT POLYOL with PX 521-522 HT ISO ISOCYANATE reactive components (Axson Technologies, Newmarket Suffolk, UK) at standard pressure and room temperature without the use of any catalysts. After five minutes of vigorous stirring with an ultrasonic mixer (WELDER, Hiroshima, Japan) in a vacuum chamber (VB, Prague, Czech Republic) to homogenize and degass the reaction mixture prior to polymerization, this mixture was hardened in the mold and left to cure at 353 ± 3 K for 4 h in a laboratory furnace XKL (France Etuves, Chelles, France). The subsequent post-curing process in XKL furnace at 373 ± 3 K took approximately 16 h. The resulting cured material was pressed in a vulcanization press machine Fontijne LabEcon 600 (Fontijne Presses, Vlaardingen, Netherlands) at a displacement speed of 2 ± 0.01 mm·min^−1^ and a force of 100 ± 2 kN. Ten rectangular shaped samples with the dimensions of 30 ± 0.3 mm (length) × 6 ± 0.06 mm (width) × 1.5 ± 0.02 mm (thickness) were cut off from the pressed TPU using a Gravograph LS100 40W CO_2_ laser cutter (Gravograph, Paris, France). 

### 2.2. DMA Testing

The DMA measurements were carried out using a Perkin Elmer Pyris Diamond 8000 Dynamic Mechanical Analyzer (Perkin Elmer, Baesweiler, Germany) in the uniaxial tensile mode. All ten prepared samples were tested at frequencies of dynamic mechanical loading of 0.5 Hz, 1 Hz, 2 Hz, 5 Hz and 10 Hz, with a constant stress value of 0.1 MPa, constant stress rate of 0.1 Hz and an amplitude of sinusoidal loading of 20 μm, over the entire temperature range of the TPU service life from 146 K up to 527 K, at a constant heating rate of 3 K·min^−1^. The strain measurements were done by a linear variable differential transformer of the DMA equipment with a span of 2 mm and a mean resolution of 2 nm. Low temperatures in the DMA measuring chamber were provided by liquid nitrogen from a 50 K Dewar flask (Perkin Elmer, Baesweiler, Germany), without direct contact of the refrigerant with the measured sample. The measurements uncertainty was about 2%. The average values of storage modulus *E′*(*T*, *f*), loss modulus *E″*(*T*, *f*), and loss tangent tan*δ* (*T*, *f*) were computed.

### 2.3. RBF-ANN Modeling

For the modeling of the experimental results of the multi-frequency DMA tests, a new multi-variable RBF-ANN model was developed. The structure of the RBF-ANN consists of an input layer, a single hidden layer with the non-linear RBF transfer functions, and a linear output layer. A Gaussian function was used as the RBF of the hidden layer neurons in the form of [32]

(1) $$\varphi_{j}\left(x_{i}\right)=\exp\left(-{\frac{\left\|x_{i}-\mu_{j}\right\|}{2\sigma_{j}^{2}}}^{2}\right),$$

where *μ_j_*, *σ_j_* and *φ_j_*(*x_i_*) denote its well-pointed center (centroid), spread width (stretch constant) and the response of the *j*-th hidden neuron corresponding to its input *x_i_*, respectively. The term

(2) $$d_{j}\left(x_{i}\right)=\left\|x_{i}\;-\;\mu_{j}\right\|,$$

expresses Euclidean distance between the elements of the input vector *x_i_* and the corresponding centroid of Gaussian *μ_j_* (Euclidean norm of RBF). 

The input data of the ANN are experimental temperatures *T* and frequencies *f*, while the target data are the corresponding average experimental values of *E′*(*T*, *f*), *E″*(*T*, *f*) and tan*δ*(*T*, *f*) for each temperature of the tested TPU samples at each given frequency of the dynamic mechanical loading. The input layer has two neurons, and the output layer has three neurons, which corresponds to the number of the input and output variables of the model. The number of hidden neurons was searched for in the process of optimizing the model with the use of trial and error method. 

The task of the input layer, consisting of one neuron for each input variable of the network, is only to feed the hidden layer 1 with the elements of the input signal matrix

(3) $$p=\left(\begin{array}{l} f^{1}\;\;f^{1}\;\ldots \;\;f^{1}\;\;\;f^{2}\;f^{2}\;\ldots \;\;f^{2}\;\ldots \;\;f^{m}\;f^{m}\;\ldots \;\;f^{m}\\ T_{1}^{1}\;\;T_{2}^{1}\;\ldots \;T_{q_{1}}^{1}\;\;\;T_{1}^{2}\;T_{2}^{2}\;\ldots \;\;T_{q_{2}}^{2}\;\ldots \;\;T_{1}^{m}\;T_{2}^{m}\;\ldots \;T_{q_{m}}^{m} \end{array}\right),$$

with the length

(4) $$R=\sum_{i=1}^{m}q_{i},$$

and size *R* × 2, composed of input data vectors f and T with the same length *R*, where *m* is the number of frequencies *f* in the multi-frequency DMA spectrum, and *q_i_* is the number of temperature samples *T_i_* for each frequency.

Prior to applying a nonlinear transformation by the RBF transfer function, each element *p_i_* of the input signal p both input variables is multiplied by the respective element *b_j_* of the hidden layer bias vector b^1^ with the length *S*^1^ corresponding to the number of hidden neurons. Each bias *b_j_* of hidden neuron *j* represents a constant external input with a value of 1 multiplied by its weight *w_ji_*, which is the appropriate element of the input weight matrix of hidden layer IW^1,1^ with the size *S*^1^x*R*, and is the primary activity level of the hidden neuron *j*. The vector n^1^ with the length *S*^1^, whose elements *n_j_* represent the distances between the multiplied inputs *p_i_* and their related weights *w_ji_*, serves as the net input n^1^ of the hidden layer, or as the activation potentials [24] of its RBF neurons.

The RBF transfer function of each neuron *j* of hidden layer 1 with the input

(5) $$n_{j}=\sum_{i=1}^{R}\;\left(\left\|p_{i}-w_{ji}\right\|\right)b_{j},\;\mathrm{for}\;j=1,\;2,\;3,\;\ldots \;,\;S^{1},$$

produces for each input variable of the model the following output [38]

(6) $$a_{j}\left(n_{j}\right)=\exp\left[\sum_{i=1}^{R}\;\left(-\frac{{\left\|p_{i}-w_{ji}\right\|}^{2}}{\sigma_{j}^{2}}b_{j}\right)\right],\;\;\mathrm{for}\;j=1,\;2,\;3,\;\ldots \;,\;S^{1},$$

which is an element of the vector a^1^ with the length *S*^1^. The weight *w_ji_* represents the centroid of the *j*-th hidden RBF neuron. The total output of nonlinear hidden layer 1

(7) $$a^{1}=\mathrm{radbas}\left(\left\|IW^{1,1}-p\right\|b^{1}\right),$$

where [38]

(8) $$\mathrm{radbas}\left(\left\|IW^{1,1}-p\right\|b^{1}\right)=\exp\left[\sum_{i=1}^{R}\;\left(-\frac{{\left\|p_{i}-w_{ji}\right\|}^{2}}{2\sigma_{j}^{2}}b_{j}\right)\right],\;\mathrm{for}\;j=1,\;2,\;3,\;\ldots \;,\;S^{1},$$

it is consequently sent to the input of the linear output layer. The connections between the hidden and the output neurons are linear weighted sums, so each neuron *k* of the output layer 2 computes the weighted sum of its inputs *a_j_* and biases *b_k_*, so

(9) $$n_{k}=\sum_{j=1}^{S^{1}}w_{kj}a_{j}+b_{k}^{2},\;\mathrm{for}\;k=1,\;2,\;3,\;\ldots \;,\;S^{2},$$

where *w_kj_* are the corresponding elements of the output layer weight matrix LW^2,1^ with the size *S*^2^ × *S*^1^, *b_k_* are the elements of the vector of biases of the output layer b^2^ with the length *S*^2^ and *n_k_* elements of the vector n^2^ with the length *S*^2^, which serves as the net input n^2^ of the output layer 2, or as the activation potential of its linear neurons.

The output value *a_k_* of the linear transfer function of *k*-th output layer neuron with the input *n_k_* for each input variable of the model is given by the expression

(10) $$a_{k}\left(n_{k}\right)=\sum_{j=1}^{S_{1}}w_{kj}\exp\left[\sum_{i=1}^{R}\;\left(-\frac{{\left\|p_{i}-w_{ji}\right\|}^{2}}{2\sigma_{j}^{2}}\right)b_{j}\right]+b_{k},\;\mathrm{for}\;k\;=\;1,\;2,\;3,\;\ldots \;,\;S^{2},$$

where *a_k_* is the element of the output layer 2 output vector [38]

(11) $$a^{2}=y=\mathrm{purelin}\left(LW^{2,1}\;a^{1}+b^{2}\right),$$

with the length *S*^2^, which is also the overall output of RBF-ANN y, and

(12) $$\mathrm{purelin}\left(LW^{2,1}\;a^{1}+b^{2}\right)=LW^{2,1}\;a^{1}+b^{2}.$$

It follows from the above that the approximation of the functional dependencies of *E′*(*T*, *f*), *E″*(*T*, *f*) and tan*δ*(*T*, *f*) realized by RBF-ANN with the described structure can be expressed as a linear combination of optimal number of Gaussian RBF neurons with adequate spread width, centered in the RBF centroids, in the form of

(13) $$y\left(p\right)=LW^{2,1}\mathrm{radbas}\left(\left\|IW^{1,1}-p\right\|\;b^{1}\right)+b^{2}.$$

However, for a given network to have good predictive capabilities, it must first be well trained on a suitably selected set of learning patterns originating from the same data distribution as *E′*(*T*, *f*), *E″*(*T*, *f*) and tan*δ*(*T*, *f*).

**Figure 1:**
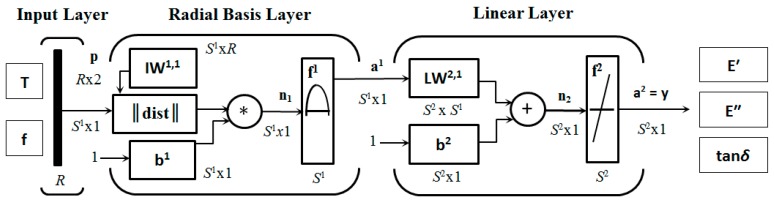
Diagram of the RBF-ANN structure for DMA multi-frequency spectrum prediction.

A scheme of the structure of created RBF-ANN is shown in Figure 1. The network was realized in the Neural Network Toolbox of the Matlab^®^ Version 9.0.0.341360 R2016a 64-bit software package (MathWorks, Natic, MA, USA) that provides number of built-in functions with sufficiently powerful and user-friendly RBF-ANNs training and simulation algorithms.

To create and train RBF-ANN as described above in the Matlab^®^ development environment, a built-in newrb function was used. The algorithm of this function adds one by one neuron with the specified width of the Gaussian RBF function spread to the hidden layer until their predetermined maximum number MN is reached, or the predetermined smallest value of mean squared error (*MSE*) goal. The iterative algorithm of simultaneous networking and training starts from an empty hidden layer. After adding a new hidden neuron, a network simulation is performed in each iteration step; the input vector with the greatest network’s *MSE* is found; and Gaussian RBF (radbas) neuron is added to the hidden layer with weights corresponding to this vector and with constant bias; the linear layer weights and biases (purelin) are redesignated to minimize the network’s *MSE*. This procedure is repeated until the network’s *MSE* falls below goal or until the maximum number of neurons MN is reached [39]. The ANN’s error criterion *MSE* for each ANN output variable is calculated as the mean squared of the errors

(14) $$MSE=\frac{1}{N}\sum_{i=1}^{N}{\left(y_{i}-y_{i}^{m}\right)}^{2}.$$

where *y_i_* are target values *E′*(*T*, *f*), *E″*(*T*, *f*) and tan*δ*(*T*, *f*), *y*^m^*_i_* represent the network response to its *T_i_* and *f_i_* input values, and *N* is the number of data points in the ANN patterns. With fixed MN and goal, the best-fit function of RBF-ANN targets for both input variables *T* and *f* was searched by trial and error method for different spread parameter values for the training set of data points.

In order to train the network, validate the found RBF-ANN model and test its performance, or test the generalization capabilities of the trained network, the input and target data were divided into training, validation and testing data sets, respectively [27]. Only four of the five available experimental DMA curves were used to train the network and validate the model found, namely *E′*(*T*, *f*), *E″*(*T*, *f*) and tan*δ*(*T*, *f*) for frequencies 0.5, 2, 5 and 10 Hz, which were randomly divided in the proportion div = 0.85:0.15, respectively. The above-mentioned ratio was sought by the trial and error method in the RBF-ANN optimization process. The experimental curves *E′*(*T*, *f*), *E″*(*T*, *f*) and tan*δ*(*T*, *f*) for the fifth available frequency (1 Hz) that were not part of training or validation data were used as a testing data set to test the generalization capabilities of the trained network.

After the best-fit function of RBF-ANN targets is estimated during the learning process, the well-trained network is simulated with validation data as well as testing data. If the differences (errors) between targets **y** and simulated outputs **y^m^** for validation, as well as testing data

(15) $$\mathrm{Err}=y-y^{m},$$

are sufficiently small, RBF-ANN has good interpolation and prediction capabilities. Otherwise, it is necessary to modify the spread parameter, or possibly the goal, MN and div parameters as well, and repeat the network training process, model validation as well as testing its performance until the optimum result.

Finding the optimal network spread parameter can be very lengthy. However, in the given case, there was no time-consuming problem. Therefore, the use of any of the nonlinear intelligent optimization methods, such as the genetic algorithm described in detail, e.g. in the work [40], was not needed to optimize the created non-linear RBF-ANN system of a well-trained network model.

Performance, regression and model error plots were used for a detailed assessment of the goodness of outputs of the RBF-ANN model. The degree of correlation between outputs and targets of RBF-ANN was calculated based on the linear correlation coefficient

(16) $$R=\frac{Cov\left(p,\;y\right)}{s_{p}s_{y}}$$

for training, validation and testing data set. The symbol *Cov*(*p*, *y*) denotes the covariance of the output and target data, while *s*_p_ and *s*_y_ are their standard deviations, respectively [39]. 

Before entering into the input layer of the network, both RBF-ANN inputs and targets were normalized to the [0, 1] range according to the relationship

(17) $$z_{\mathrm{norm}}=\frac{z-z_{\min}}{z_{\max}-z_{\min}},$$

where **z** represents the original data before normalization, **z_min_** and **z_max_** are their minimum and maximum values before normalization, and **z_norm_** are data after normalization. With the applied Gaussian RBF activation functions of the hidden layer, such an adjustment of the input and target data to the interval [0, 1] is able to ensure fast and accurate convergence of network output data to the values of the analyzed experimental data. The reverse of network outputs normalized to original data after RBF-ANN simulation with training, validation and testing data was realized according to the relationship [27]

(18) $$z=z_{min}+z_{norm}\left(z_{max}-z_{min}\right).$$

## 3. Results and Discussion

### 3.1. Results of Dynamic Mechanical Analysis

**Figure 2:**
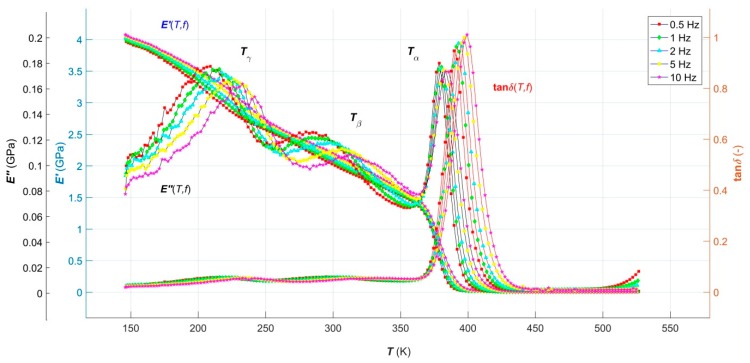
Temperature-frequency dependences of average storage modulus, loss modulus and loss tangent of TPU over the temperature range from 146 K to 527 K at constant strain rate of 0.1 Hz and frequencies of 0.5 Hz, 1 Hz, 2 Hz, 5 Hz and 10 Hz.

Results of the realized DMA tests in the form of temperature dependence of average storage modulus *E′*(*T*, *f*), loss modulus *E″*(*T*, *f*) and loss tangent tan*δ*(*T*, *f*) in a temperature range *T* from 146 K up to 527 K at external dynamic mechanical load frequencies *f* = 0.5, 1, 2, 5 a 10 Hz and a constant strain rate of 0.1 Hz are shown in Figure 2.

It can be seen from Figure 2 that the shape of the individual curves of the multi-frequency DMA spectrum of the investigated TPU corresponds to the shape of the DMA curves *E′*(*T*, *f*), *E″*(*T*, *f*) and tan*δ*(*T*, *f*) of the high molecular weight, weakly semi-crystalline TPEs below the flow region [14]. Crystalline hard segments, soft crystalline segments and amorphous soft segments phase of TPU multi-phase morphology are manifested by the presence of *α*-, *β*- and *γ*- relaxation transitions at temperatures *T*_α_, *T*_β_ and *T*_γ_, respectively, which are particularly pronounced on the curves *E″*(*T*, *f*). The temperature *T*_γ_ corresponds to the glass transition of crystalline soft segments from stiff glassy stage to compliant rubbery stage; the temperature *T*_α_ is associated with the glass transition of crystalline hard segments due to the breakdown of hydrogen-bonded interactions of van der Waal’s forces between the rigid and flexible segments of TPU, while at the temperature *T*_β_, the short-range order translation, and reorientation motions within both the soft-phase and hard-phase crystallites, occur [15]. Activation energies of individual relaxation transitions with values of 379.73 kJ·mol^−1^ for *α*-transition, 65.53 kJ·mol^−1^ for *β*-transition and 45.98 kJ·mol^−1^ for *γ*-transition, calculated based on the linearized Arrhenius equation from the frequency-dependent shift of transition temperatures taken as peaks of tan*δ*(*T*, *f*) in our earlier work [17], confirm that at temperature *T*_α_ the primary relaxation transition and at temperatures *T*_β_ and *T*_γ_ the secondary relaxation transitions actually occur [16].

A significant phase separation level, induced by the symmetrical structure of rigid segments, a low level of crosslinking and a small degree of TPU crystallinity, are documented by a sharp drop of *E′*(*T*, *f*) curves in its glassy region. The rapid slope of the glass transition of the rigid segments, observed on the tan*δ*(*T*, *f*) curves, are caused by the homogeneous structure of the physical network and by the narrow variance of the molecular weights. The broad and virtually temperature independent rubbery plateau is the result of uniform rigid segments forming stable crystallites, which soften in a relatively narrow temperature range, as is evident from the increase in tan*δ*(*T*, *f*) curves at the end of the DMA multifrequency spectrum [17,21]. With increasing the frequency of dynamic mechanical loading, the transition temperatures of all three observed relaxation events shifted to higher values, which means that the studied TPU acts in a more and more elastic fashion with an increasing frequency. In higher frequencies, the macromolecular segments are not able to react to the dynamic mechanical load during each cycle by a corresponding change in its conformation as in the case of lower frequencies, or higher time delays. Therefore, the dissipation of mechanical energy was lower, as a result of which the TPU acted as more elastic at higher frequencies, or it exhibited its elastic properties with higher storage modulus, lower loss modulus, and lower loss tangent [14,17].

Unfortunately, there is currently no analytical model capable of predicting viscoelastic behavior of polymeric systems over their entire temperature lifetime range and with a sufficiently broad frequency of their dynamic mechanical load, including all relaxation transitions that occur, with a sufficiently high level of reliability. The presented work is a contribution aiming to remove this deficit by creating a new single RBF-based ANN model of temperature-frequency dependence of all three *E′*(*T*, *f*), *E″*(*T*, *f*) and tan*δ*(*T*, *f*), the analysis of which is given in the following paragraph.

### 3.2. Analysis of the RBF-ANN Model

Created ANN for functional dependencies *E′*(*T*, *f*), *E″*(*T*, *f*) and tan*δ*(*T*, *f*) provides results with the lowest *MSE* and the highest accuracy *R* of all three training, validation and testing data sets simultaneously at 268-Gaussian RBF neurons. Thus, the optimal RBF-ANN model for describing and predicting these thermo-mechanical parameters can be presented in the form of 2-268-3 (number of input-hidden-output layer neurons) [23]. 

The network parameters obtained in the optimization process are summarized in Table 1, the meaning of which is as follows: IL - number of neurons in the input layer; HL - number of neurons in the hidden layer; OL - number of neurons in the output layer; TF - activation function of hidden and output layer neurons, respectively; DDF - function of dividing input-target data into training and validation data; PF - network error calculation function; MN - maximum number of neurons in the hidden layer; spread – neuron’s spread width; goal - the smallest *MSE* target value; RBF - radial basis transfer function (radbas); linear - linear transfer function (purelin); MSE - mean squared error; dividerand - random division of input-target data.

As mentioned above, performance, regression and model error plots were used for a detailed assessment of the goodness of an ANN model.

**Figure 3:**
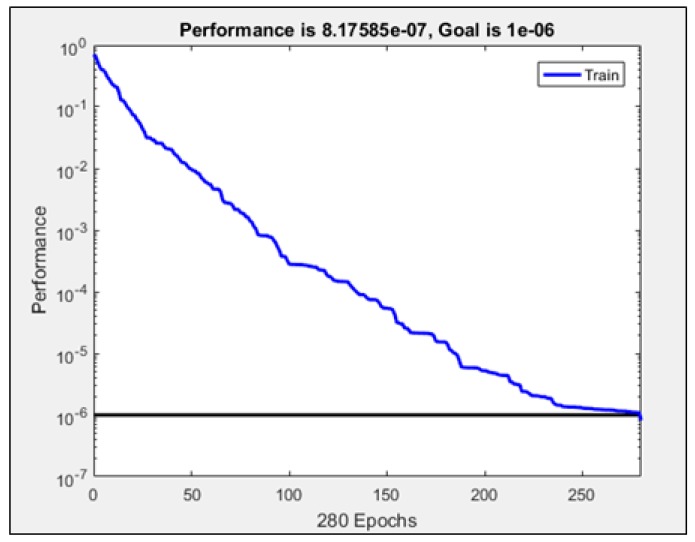
Performance plot of training RBF-ANN.

A performance plot of RBF-ANN that shows how *MSE* is minimized during the network training process is shown in Figure 3. It is clear from the figure that *MSE* of training data decreases continuously during learning without any indication of over-fitting or under-fitting. The best performance of the model occurs in the last 268th epoch of the learning iteration cycle, with an associated final *MSE* of approximately 8.176 × 10^−7^, which is significantly lower than the target *MSE* value of 1 × 10^6^, indicating that the best-fit function of the training targets was perfectly estimated.

**Figure 4:**
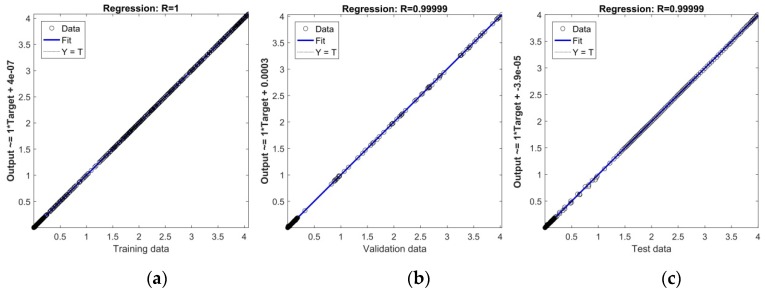
(**a**) Linear regression plot for training data; (**b**) Linear regression plot for validation data; (**c**) Linear regression plot for testing data.

While *MSE* function is a performance metric adopted to determine the ANN performance, *R*-value measure the correlation between ANN outputs and ANN targets. In Figure 4a–c, the linear regression plots of targets relative to outputs (fitness plots) for all three training, validation and testing data sets used are presented, respectively. From these figures, it is obvious that practically all data (hollow circles) fall on the 45° line with the equation

(19) Output ~=slope⋅Target+intercept,

where *slope* = *R*, expressing the exact match between outputs and targets with *R* equal or very close to 1, which indicates excellent descriptive, interpolation, as well as predictive abilities of the created model. The results of the training process, validation and testing of the created RBF-ANN model, along with the corresponding ANN-optimized parameters, are summarized in Table 2.

**Figure 5:**
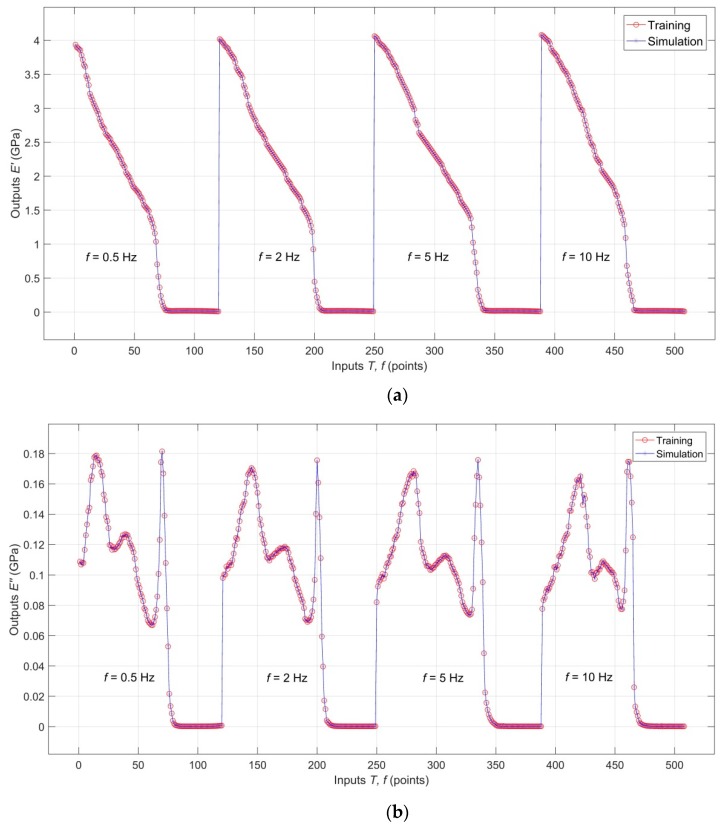

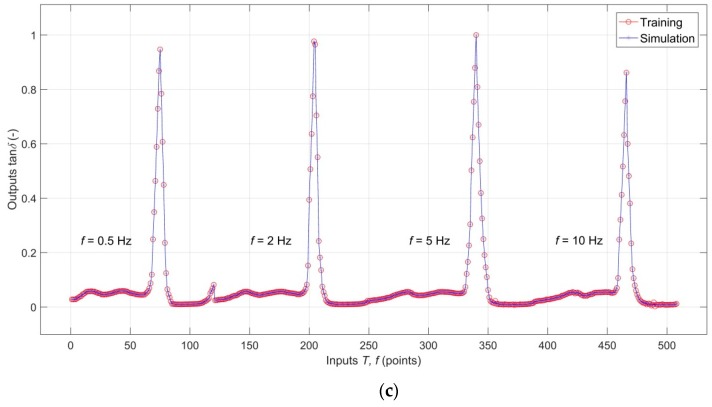
(**a**) Comparison of the training target with simulated training outputs for *E′*(*T*, *f*); (**b**) Comparison of the training target with simulated training outputs for *E″*(*T*, *f*); (**c**) Comparison of the training target with simulated training outputs for tan*δ*(*T*, *f*).

A comparison of training targets with simulated training outputs of the network for *E′*(*T*, *f*), *E″*(*T*, *f*) and tan*δ*(*T*, *f*), documenting perfect goodness of fit-to-targets, is shown in Figure 5a–c, respectively.

**Figure 6:**
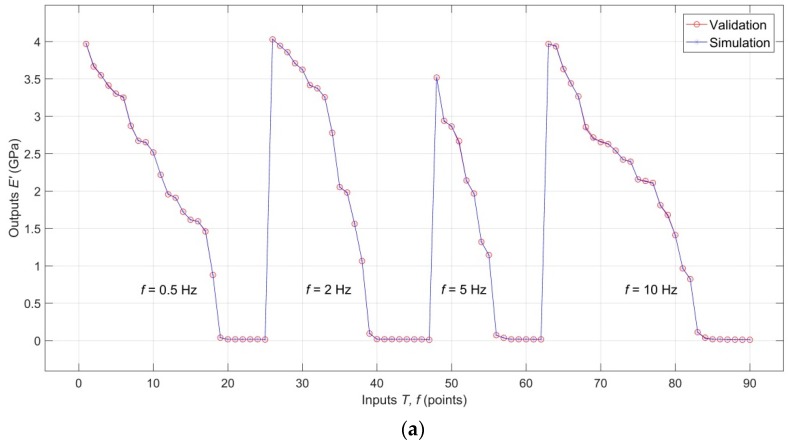

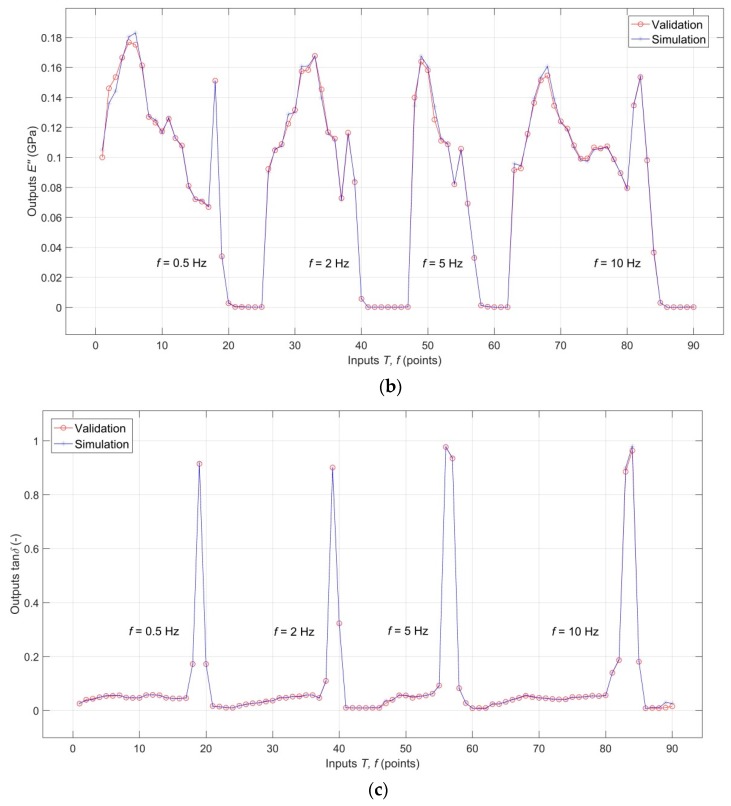
(**a**) Comparison of the validation targets with validation outputs for *E′*(*T*, *f*); (**b**) Comparison of the validation targets with validation outputs for *E″*(*T*, *f*); (**c**) Comparison of the validation targets with validation outputs for tan*δ*(*T*, *f*).

**Figure 7:**
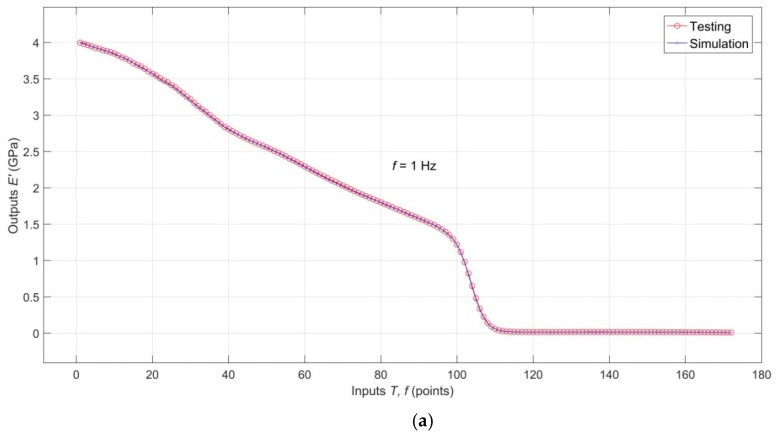

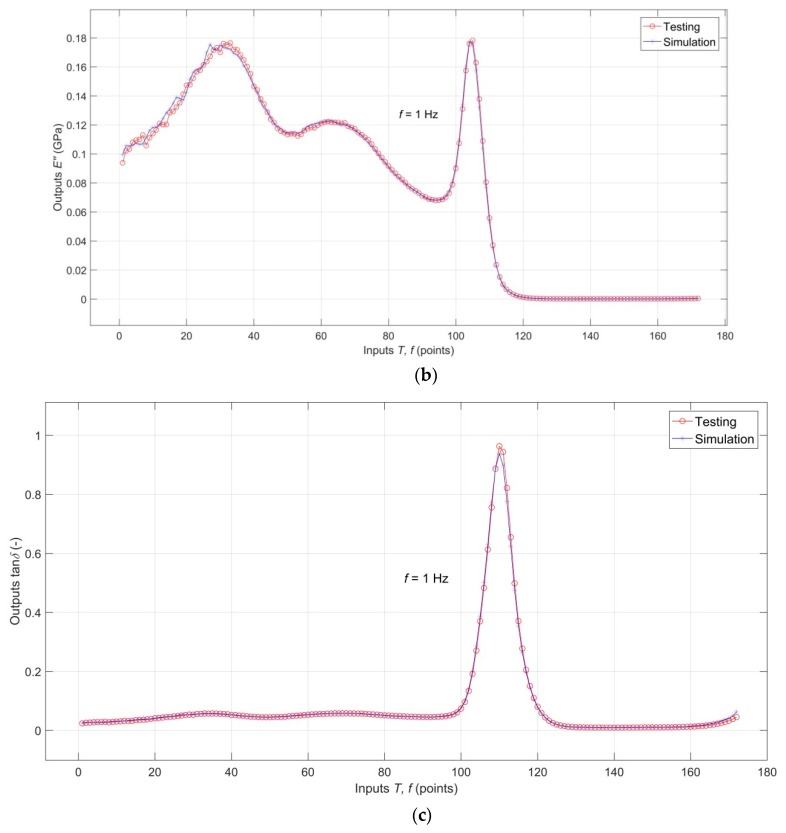
(**a**) Comparison of the testing targets with testing outputs for *E′*(*T*, *f*); (**b**) Comparison of the testing targets with testing outputs for *E″*(*T*, *f*); (**c**) Comparison of the testing targets with testing outputs for tan*δ*(*T*, *f*).

Comparison of the validation targets with validation outputs for *E′*(*T*, *f*), *E″*(*T*, *f*) and tan*δ*(*T*, *f*), documenting excellent interpolation capabilities of the model, is shown in Figure 6a–c, respectively. An analogical comparison for testing targets and testing outputs is shown in Figure 7a–c, which shows that the analyzed RBF-ANN model, in addition to excellent descriptive and interpolation capabilities, also has very good predictive capabilities.

**Figure 8:**
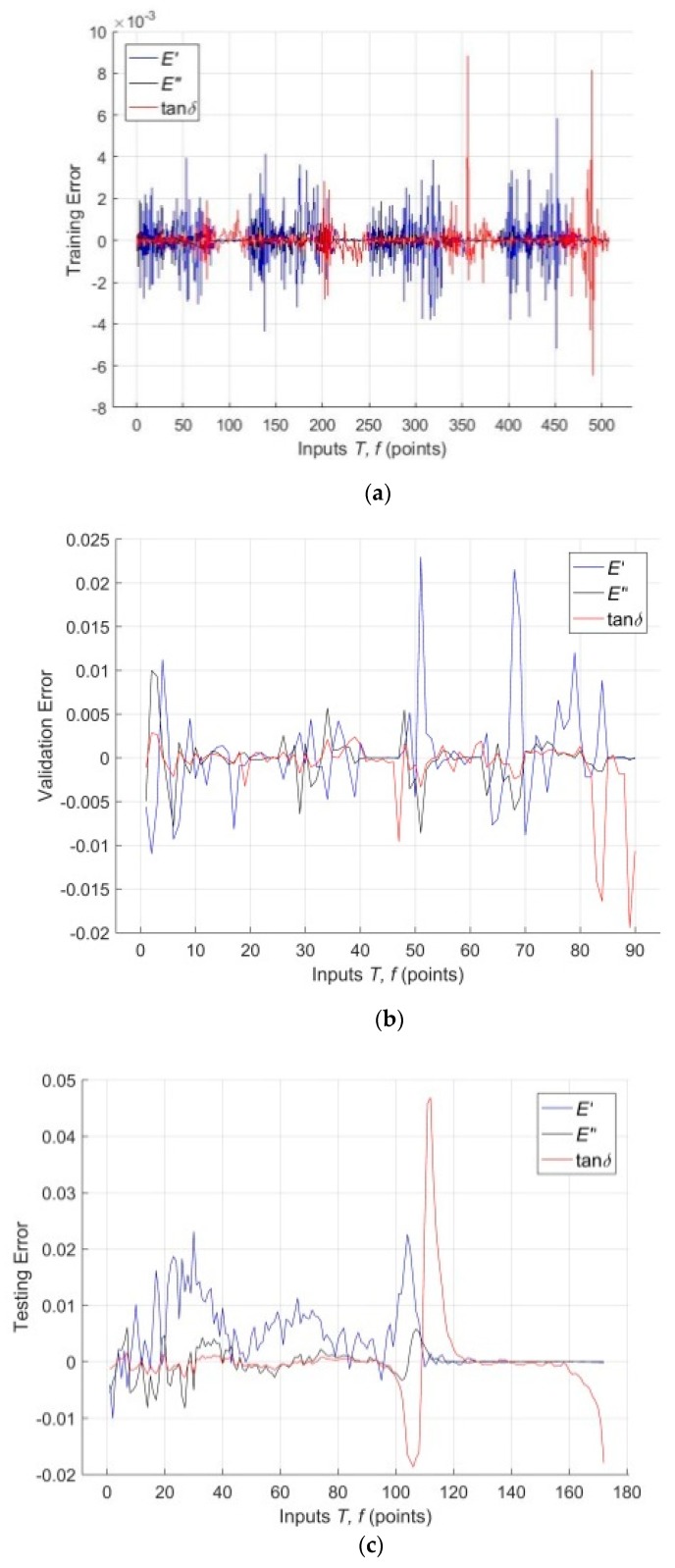
(**a**) Error plot for training data; (**b**) Error plot for validation data; (**c**) Error plot for testing data.

The differences between training, validation as well as testing targets and outputs for *E′*(*T*, *f*), *E″*(*T*, *f*) and tan*δ*(*T*) in a graphical form (model error plots) are presented in the Figure 8a–c. It is clear from these figures that the error at the order of 10^−2^ is negligibly small compared to the experimental data acquired from the DMA tests. The maximum training data error of 0.87% is shown by tan*δ*(*T*), validation data at 0.56 % *E′*(*T*, *f*) and testing data at 4.63 % tan*δ*(*T*), which can be considered an excellent result.

Since tan*δ*(*T*, *f*) is the ratio of *E″*(*T*, *f*) to *E′*(*T*, *f*), the three target values in training ANN are not totally independent. That means the above-described RBF-ANN may be only created with two output neurons for *E″*(*T*, *f*) and *E′*(*T*, *f*). Training, validation and testing of such a modified network showed that the number of hidden neurons of the optimal model decreased from 268 to 219 and the training time from approximately 22 to 17 s, i.e., approximately about 18% and 23%, respectively. Still, the linear correlation coefficient *R* has not changed for training and validation data, while its value for the test data has risen from 0.99999 to 1. Thus, the optimized RBF-ANN model 2-219-2 has a slightly higher computational efficiency compared to the model 2-268-3, which, however, has practically no effect on the overall model error that remains on the same level. 

Whereas the multi-frequency DMA spectrum for all TPEs has an analogous shape, it can be expected that the temperature dependencies of their dynamic thermo-mechanical parameters over their entire temperature lifetime range and in a wide range of external dynamic mechanical load frequencies can be very efficiently modeled with a high level of reliability through RBF-based ANN. However, the area of the excellent predictive performance of the RBF-ANN model is essentially limited to just the width of the interval of the input-target patterns learned by the network, which is a consequence of the relatively poor extrapolation capabilities of ANNs in general (as opposed to their excellent interpolation performance) [26]. ‘However, the area of the excellent predictive performance of the RBF-ANN model is essentially limited to just the width of the interval of the input-target patterns learned by the network, which is a consequence of the relatively poor extrapolation capabilities of ANNs in general (as opposed to their excellent interpolation performance) [26]. The prediction of temperature dependencies *E′*, *E″* and tan*δ* of TPEs below the temperature *T_δ_* and above the temperature *T*_f_ makes no sense in practical terms, since this temperature range includes the whole temperature range of their lifetime and is usually the standard temperature interval of DMA measurements of TPEs. Frequently, however, it is necessary to know the values of *E′*, *E″* and tan*δ* at temperatures that are not between the experimental data although they come from the measured temperature interval. ANNs’ excellent interpolation capabilities make it possible to solve this problem.

On the other hand, the dynamic mechanical stress of the polymeric materials in various practical applications is realized in an extremely wide frequency range. Therefore, the prediction of temperature dependencies *E′*, *E″* and tan*δ* for different frequencies is of great practical importance, especially considering that multi-frequency DMA measurements are extremely time-consuming. The reliability of such prediction is determined by the optimal number of representative experimental data selected as input-target patterns for network training, as well as by their quality. In general, the smaller the spacing between the individual frequencies of the DMA spectrum, the higher the predictive performance of the network across the measured frequency interval can be expected to be.

The results of DMA measurements are influenced by a number of internal and external factors (sample size, stress value, strain rate, heating rate and other factors [15]) that can be added to the network as additional input parameters prior to training. The measurements to get the results presented in our work were performed in full compliance with the DMA manufacturer’s instructions and the relevant standard for polymer materials, however, the effect of these factors on temperature and frequency dependence of the thermo-mechanical parameters in question has not been observed and may be of interest for ongoing research.

## 4. Conclusions

In the present work, the new radial basis function artificial neural network-based model of dynamic thermo-mechanical response and damping behavior of thermoplastic elastomer systems in the whole temperature range of their lifetime and a wide frequency range of dynamic mechanical loading has been developed. The variations of storage modulus, loss modulus and loss tangent with temperature and frequency, acquired from the dynamic mechanical analysis of thermoplastic polyurethane, have been modeled by a single model of a well-trained radial basis artificial neural network with Gaussian radial basis function as activation function of hidden layer neurons and the linear transfer function of output neurons. The excellent agreement between experimental and modeled data, including all observed relaxation transitions, has been found over the entire monitored temperature and frequency range. The radial basis function artificial neural network has been confirmed to be an exceptionally high-performance artificial intelligence tool of soft computing for the effective predicting of short-term viscoelastic behavior of thermoplastic elastomer systems based on experimental results of dynamic mechanical analysis tests.

## Figures and Tables

**Table 1:** RBF-ANN parameters for an optimized model of temperature-frequency dependence of dynamic thermo-mechanical parameters *E′*(*T*, *f*), *E″*(*T*, *f*) and tan*δ*(*T*, *f*) of TPU.

IL	HL	OL	TF	DDF	PF	MN	Spread	Goal
2	268	3	Gaussian RBF, linear	dividerand	MSE	10^3^	7	10^−6^

**Table 2:** Parameters of optimized ANN and results of the training network process, validation and testing of the RBF-ANN model.

	Data Division	Samples	MSE	R	Intercept
Training	0.85	1016	8.176 × 10^−7^	1	4.9 × 10^−7^
Validation	0.15	180	-	0.99999	3 × 10^−4^
Testing	1	344	-	0.99999	3.9 × 10^×5^
